# Modeling autism: a systems biology approach

**DOI:** 10.1186/2043-9113-2-17

**Published:** 2012-10-08

**Authors:** Mary Randolph-Gips, Pramila Srinivasan

**Affiliations:** 1 Systems Engineering and Computer Engineering, University of Houston – Clear Lake, 2700 Bay Area Bvd, Houston, TX, 77058, USA; 2 MedicalMine Inc, 5611 Highland Road, Pleasanton, CA, 94588, USA

**Keywords:** Autism, Mitochondrial dysfunction, Oxidative stress, Immune dysfunction, Gastrointestinal disease

## Abstract

Autism is the fastest growing developmental disorder in the world today. The prevalence of autism in the US has risen from 1 in 2500 in 1970 to 1 in 88 children today. People with autism present with repetitive movements and with social and communication impairments. These impairments can range from mild to profound. The estimated total lifetime societal cost of caring for one individual with autism is $3.2 million US dollars. With the rapid growth in this disorder and the great expense of caring for those with autism, it is imperative for both individuals and society that techniques be developed to model and understand autism. There is increasing evidence that those individuals diagnosed with autism present with highly diverse set of abnormalities affecting multiple systems of the body. To this date, little to no work has been done using a whole body systems biology approach to model the characteristics of this disorder. Identification and modelling of these systems might lead to new and improved treatment protocols, better diagnosis and treatment of the affected systems, which might lead to improved quality of life by themselves, and, in addition, might also help the core symptoms of autism due to the potential interconnections between the brain and nervous system with all these other systems being modeled. This paper first reviews research which shows that autism impacts many systems in the body, including the metabolic, mitochondrial, immunological, gastrointestinal and the neurological. These systems interact in complex and highly interdependent ways. Many of these disturbances have effects in most of the systems of the body. In particular, clinical evidence exists for increased oxidative stress, inflammation, and immune and mitochondrial dysfunction which can affect almost every cell in the body. Three promising research areas are discussed, hierarchical, subgroup analysis and modeling over time. This paper reviews some of the systems disturbed in autism and suggests several systems biology research areas. Autism poses a rich test bed for systems biology modeling techniques.

## Background

Autism is the fastest rising developmental disorder in the world today. In the US the rates of autism have risen from 1 in 2500 in the 1970 [1] to 1 in 88 today [2]. Autism is defined behaviorally, and is characterized by impairments in social behavior, stereotypic movements and difficulties in communicating [3]. Autism presents a burden upon both families and society as a whole. The estimated total lifetime societal cost of caring for one individual with autism is $3.2 million US dollars. This includes direct costs such as medical, therapeutic, educational and child and adult care. This figure also includes indirect costs such as loss of productivity of both the individual with autism and their caregivers [4].

In the past autism was considered purely a psychological [5] or neurological disorder [6]. There is increasing evidence that it is a highly diverse disease affecting multiple systems of the body. Some systems with strong evidence of involvement are metabolic, gastrointestinal, immunological, mitochondrial, and neurological [7,8]. Identification and modeling of these systems may lead to new treatments. It is hard to predict the all new treatments that would result from a systems approach, but the first would be better targeting of treatments. At present, physicians often rely on therapeutic trials and on psychotropic drugs not approved for autism [9].

One of the difficulties in describing the biology of autism is that it appears to have multiple etiologies. Some children have gastrointestinal disease, while others do not [10]. Some children have frank immune disorders, while others appear healthy [11]. Some show signs of autism from birth, while others appear to have a period of normal development, and then regress [12]. In addition to the difficulties this presents for modeling autism, the complex etiology can be a confounding factor in many autism studies as the different subgroups are not apparent using just the defining behavioral characteristics.

Currently those going in for autism evaluation do not get a comprehensive workup. These patients cannot articulate their problems or have the cognition to request an evaluation, so we need better lab workups. Many of these patients present with behavioral challenges, so the testing procedures should be all-encompassing and as less invasive as possible. So this whole body approach to modeling could potentially generate the parameters for a comprehensive evaluation or intake that would best guide treatment.

Another difficulty in understanding autism is that the various systems involved interact in complex and highly interdependent ways. This complexity points to a new paradigm in autism research using systems biology. In addition, autism poses particular difficulties as the scale of information to be modeled varies widely, from molecular level to anatomical. The diverse systems involved in autism and its complex etiology, makes the development of new techniques to model autism and mine its data, imperative. This paper will first review the systems that are altered in people with autism, and then present some of the challenges autism presents to system biologists.

### Genetics, metabolism and oxidative stress

Autism has an established genetic component. Studies of twins shows a concordance of 0-10% in dizygotic twins and 70-90% in monozygotic twins [13,14]. However, the search for single autism genes has not been fruitful. It appears that autism results from a combination of relatively high frequency genes. The current model predicts that between 10 and 100 possible genetic variants may be responsible [15]. The rising rates of autism and the fact that the concordance of identical twins is not 100% supports the theory that autism results from a combination of genetic and environmental factors [16-18].

Several genetic variants have been associated with increased risk for autism. The variants found so far are mostly associated with differences in the metabolism, rather than in brain structure. The *MET* promoter variant rs1858830 allele “C”, found at increased rates in autism, is associated with neuronal growth and development, but also is involved in immune function and gastrointestinal repair [19,20]. The fact that this genetic variant is present in 47% of the general population gives credence to the assertion that there is an environmental component to the development of autism. Many of the genetic variants at increased prevalence in autism are associated with the folic acid, transmethylation and transsulfuration metabolic pathways. Some of these genes are MTHFR, COMT, GST, RFC and TCN2. As with the MET variant, these are common in the general population. These variants decrease the activity of enzymes and decrease the efficiency of the body’s ability to resolve oxidative stress, methylate genes and detoxify exogenous and endogenous toxins [21].

**Figure 1:**
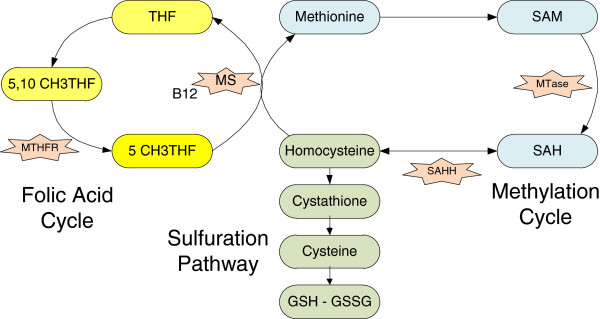
**Metabolic pathway diagram of the main cycles in the detoxification pathways.** Metabolic products are indicated by ovals and relevant enzymes by stars. Flux can be either one way, indicated by single arrow lines, or reversible, indicated by double arrow lines.

Oxidative stress occurs when production of Reactive Oxygen Species (ROS) and Reactive Nitrogen Species (RNS) exceeds the body’s ability to neutralize them. ROS/RNS are free radicals, highly reactive molecules which can damage many parts of the cell. ROS/RNS occur through the energy production process in the mitochondria and through environmental sources. The mitochondrion is the main source of ROS/RNS and has evolved a system to neutralize the oxidants. The most important among these defences is glutathione (GSH). If the mitochondrial GSH pool is low, increased mitochondrial ROS production can occur. GSH is also the main antioxidant for extra-mitochondrial parts of the cell. GSH is produced by the sulfuration pathway as shown in Figure 1. The sulfuration pathway is linked to the methylation and folic acid pathways and any perturbation of those pathways will affect the production of GSH.

The methylation pathway provides methyl groups, CH3, to many functions in the body. S-adenosylmethionine (SAM) transfers methyl groups to be used in over 150 methyltransferase dependant methylation reactions in the body [22], most notably the methylation of genes. This transfer results in S-adenosylhomocysteine (SAH). SAH can be reversibly transformed into homocysteine and adenosine by the SAH hydrolase (SAHH). Homocysteine can then be either remethylated to methionine or can be transferred to the sulfuration pathway to create glutathione. The pathway flux is influenced by the relative amounts of the components. If the activity of methionine synthase (MS) is reduced, either through availability of its cofactor cobalamin (vitamin B12) or other impairment, less homocysteine will be converted to methionine to continue the cycle. This will result in more homocysteine and SAH, which reduces SAM dependent methylation processes. Methylation serves many important functions in the body. It is used epignetically to turn on and off genes. A methylated gene will not be expressed [23]. Methylation is also important in the function of neurotransmitters, neurohormones, myelin, membrane phospholids, proteins and creatine [24].

The activity of MS also determines the proportion of homocysteine shunted into the sulfuration pathway to make GSH. As the MS cofactor cobalamin is easily oxidized, oxidative stress will cause more homocysteine to be turned into GSH. In a properly functioning system this additional GSH would resolve the oxidative stress. But in autism there is evidence of continued oxidative stress [25].

Metabolic markers of oxidative stress have been found to be elevated in children with autism. Glutathione, the main cellular antioxidant, levels were reduced. In addition, the oxidized disulfide form of glutathione (GSSG) was increased resulting in a doubling of the GSSG/GSH ratio, The ratio of plasma S-adenosylmethionine (SAM) to S-adenosylhomocysteine (SAM/SAH ratio) was reduced [26,27]. Evidence of increased lipid peroxidation was found which might indicate oxidative stress [28]. Oxidative stress can have a negative effect on many systems in the body. It has been implicated in cancer, cardiovascular disease, and autoimmune disease [29-31]. Oxidative stress is particularly destructive to the brain. The brain has higher energy requirements, high concentration of polyunsaturated fatty acids and lower reserves of GSH. Oxidative stress is also increased in schizophrenia, bipolar disorder and Parkinson’s disease [32-35].

These interacting cycles are of great importance in autism as they have the potential for therapeutic intervention. Defects in MTHFR enzyme can be bypassed by supplementing the 5-CH3THF form of folic acid. Supplemental cobalamin can increase the efficiency of MS [36]. Supplements of other enzyme cofactors might also be of benefit [7].

The impairment of the metabolic pathways in autism can result from environmental influences in addition to genetics. Heavy metals [22] and pesticides [37,38] have been shown to inhibit the enzymes often deficient in autism. This could form a feedback loop, where insufficient activity of these systems allows toxins to remain, where they can further impair the detoxification systems.

### Mitochondrial system

Mitochondria are the organelles responsible for the energy production in most eukaryotic cells. They convert the energy from carbohydrates and fats into adenosine triphosphate (ATP) through the process of cellular respiration. ATP is used to power most cellular functions. Mitochondria are also involved in signalling, cellular differentiation, and apoptosis, as well as the control of the cell cycle and cell growth [24].

The mitochondrion utilizes a complex series of chemical reactions to produce the ATP. During this process free radicals, including the particularly damaging super oxide, are produced. Since free radicals are so destructive, the mitochondrion has a series of defences to reduce the free radicals. If, due to genetic defects or acquired dysfunction, more free radicals are produced than the defences can reduce, oxidative stress can occur [39]. Mitochondrial disease occurs when there are mutations in the mitochondrial DNA. Mitochondrial disease is associated with a multitude of disorders including hypotonia, mitochondrial encephalomyopathy, cardiomyopathy and a range of endocrine, hepatic or renal tubular dysfunctions, myoclonic epilepsy and mitochondrial myopathy and developmental delay among others. Mitochondrial disease has many different presentations as a child can inherit a mixture of normal and mutated mitochondria from the mother [40].

There is clinical evidence of mitochondrial disease and dysfunction in autism [41]. Although only a small minority of people with autism have mitochondrial DNA (mtDNA) mutations, the rate of autism is higher among children with mitochondrial disease [42-46]. In addition, the task of finding genetic mutations influencing mitochondrial function is confounded by the fact that many mitochondrial functions are encoded by nuclear DNA.

Mitochondrial dysfunction occurs when there is reduced mitochondrial function without genetic changes. Mitochondrial dysfunction and oxidative stress has been implicated in a variety of neurodegenerative diseases such as Alzheimer’s disease (AD), Parkinson’s disease (PD), amyotrophic lateral sclerosis (ALS) and Huntington’s disease (HD). Since the brain has high energy demands, it is more susceptible to damage from faulty mitochondria [47]. Mitochondria can be inhibited by many stressors, but chief among them are metals such as mercury, arsenic, cadmium and lead [48,49]. Pesticides and industrial chemicals have been found to inhibit mitochondrial function [50]. In addition, people with autism have been found to have higher levels of the bacterium clostridium in their guts. Clostridium produces proprionic acid, which inhibits the oxidative phosphorylation of the mitochondria [8].

Although most people with autism have no discernible mutation indicating primary mitochondrial disorder, labwork gives evidence to reduced mitochondrial function, namely elevated plasma lactate, hyperlactacidemia and increased lactate/pyruvate ratio. Rarely have mtDNA changes been found in people with autism with clinical signs of mitochondrial dysfunction [51-53]. In addition, levels of enzymes associated with resolving mitochondrial produced radical production have been found to be lower in people with autism [54].

In addition to producing ATP, mitochondria perform the important function of sequestering calcium. Calcium is also used as a biologic signal between the mitochondria and the endoplasmic reticulum. Neuronal calcium signalling causes the release of neurotransmitters and can affect the speed of signals. Diseases with defects in the mitochondrial calcium pathways have a high Co-morbid occurrence of autism [55]. Post mortem studies of autistic brains show alterations in calcium homeostasis. This study also showed a possible connection between ionized calcium levels and the immune system [56].

There are several pathways for impaired mitochondrial function to affect the brain. The brain has high energy demands and a limited ability to neutralize free radicals, thus impaired mitochondria might be damaging to neurons [57]. Mitochondrial dysfunction could also lead to reduced frequency of neuron firing, particularly of inhibitory neurons [58]. Mitochondrial dysfunction could also affect the brain indirectly, through the immune system. Mice with mitochondrial deficiency have reduced number of immune cells [59], and supplementation of mitochondrial nutrients improve immune function of Type 2 diabetic rats [60]. Mitochondrial dysfunction in areas outside the brain could lead to hepatic production of VLCFA-containing lipids arising from impaired mitochondrial fatty acid beta-oxidation. These lipids can lead to microglial activation, and release of the neurotoxin glutamate [61].

### Immune system

There is strong evidence of immune dysfunction in children with autism. Relatives of children with autism have increased rates of autoimmune diseases [62]. Imbalances of immune system cells and cytokines are found in many different parts of the immune system of people with autism.

Total levels of lymphocytes are reduced [63,64]. The serum immunoglobulin subtypes show abnormal patterns. In particular there is often a skewed Th1-Th2 helper ratio, with most people with autism showing a Th2 predomination [64,65]. T2 skewing results in increased antibodies which can induce allergies and autoimmune reactions. Food allergies are common in children with autism [66]. Th2 skewing also makes chronic viral infections more likely. Skewing also occurs in the serum immunoglobulin subtypes. Immunoglobulins are antibodies formed by the B cells to create humoral, persistant immunity. Immunoglobulins IgM, IgA, and total IgG are depressed while IgG subtypes IgG2 and IgG4, and total IgE are increased [67-70]. Increases of pro-inflammatory cytokines along with reductions of regulatory cytokines have been found [71].

The immune system has the ability to affect the mitochondria. Cytokines such as TNF*α* and IL6 can facilitate calcium influx and contribute to mitochondrial dysfunction possibly contributing to the deficits of autism through the mitochondrial system [72]. Extracellular mitochondrial DNA and anti-mitochondrial antibodies have been found in the serum of children with autism [73].

There are several avenues for the immune system to induce autistic behaviors. Immune dysregulation could result in generalized inflammation in the brain [74]. Inflammation in the brain has been linked to a number of psychiatric diseases including schizophrenia, [75] bipolar [76] Alzheimer's disease [77] and depression [78]. Multiple studies have found a correlation between abnormal levels of immune factors and core autistic deficits such as speech, mood and social deficits [79-84]. Another study found that the more the levels of the cytokines IL-1, IL5, IL-8 and IL-12p40 deviated from the norm, the more severe the stereotypical behaviors [85].

Challenge with nasal allergens during the low pollen winter months resulted in regression in 55% of children with autism as measured by the Aberrant Behavior Checklist [86]. Children with autism have been reported to have fewer aberrant behaviors particularly speech during fever as reported in a prospective study [87]. This gives further support to an immunological component [88].

The interaction between the immune system and the brain can present in several variations. Neuropeptides can modulate the immune system by recruitment of the innate immune system and chemotaxis [11]. In mouse models, decreased lymphocytes result in impaired learning and memory [89]. Autoimmunity is present in some cases. Anti-brain antibodies have been found in children with autism, though no evidence of demyelination has been found [11]. A study of 93 children with autism found that 75% had autoantibodies to the folate receptors in the central nervous system (CNS). Impairments of these receptors can lead to reduced levels of folate in the CNS and Cerebral Folate Deficiency (CFD). The levels of folate receiver antibodies were highly correlated with cerebrospinal fluid 5-methyltetrahydrofolate concentrations, thus indicating possible CFD in the tested children. There are structural similarities between the folate receptors and proteins found in milk [90]. A milk free diet, in addition to high dose folinic acid supplementation has been found to decrease the autoantibody titer and improve functioning in younger patients [91,92]. These immunological differences point to treatment options. Replacement of deficient lymphocytes in mice resolved the learning and memory difficulties [89]. Treatment of allergies often results in improvement in autistic behaviors such as hyperactivity and irritability [66]. An early study found that treatment with intravenous immune globulin in ten children with autism resulted in better speech, eye contact, focus and awareness of surroundings [93].

### Gastrointestinal system

Incidence of gastrointestinal (GI) disease among those with autism varies widely, depending on exclusion criteria and whether the study was prospective or retrospective. A prospective study showed GI symptoms in 80% of patients with autism [94]. These symptoms include abdominal pain, chronic diarrhea and or constipation, and gastro esophageal reflux disease [10]. GI disease has been confirmed via endoscopy in several studies [95-97]. Inflammation was found throughout the GI tract, with reflux esophogitis, stomach inflammation, duodenum and abnormal carbohydrate digestive enzyme activity. Other studies have found chronic patchy inflammation and lymphonodular hyperplasia. This is different than the pattern seen in classical inflammatory bowel disease, with infiltration of T cells and plasma cells into the epithelial layers of the mucosa [68,97]. Lymphocyte infiltration into the epithelial layers of the gut lining and crypt cells has been found on endoscopy. In addition, there were IgG antibodies deposited onto the epithelium and complement immune system activation. This might be indicative of an autoimmune process [98].

There is evidence of increased intestinal permeability in people with autism [99-103]. Increased intestinal permeability was even found in 43% of children with autism without clinical signs of bowel dysfunction [101]. Intestinal permeability allows larger molecules that would normally stay in the gut to cross into the bloodstream. Plasma and urinary concentrations of oxalate were greatly elevated in children with autism, which may be a result of increased intestinal absorption [104]. Increased permeability can lead to allergy and autoimmune processes. There appear to be multiple reasons for the increased permeability. The dietary protein gluten can bind to the CXCR3 receptor, resulting in increased zonulin levels. Zonulin regulates the opening of the tight junctions in the gut [105]. Ingested toxins such as Polychlorinated Biphenyls can also open the tight junctions in the gut [106].

Increased incidence of dysbiosis, an imbalance of intestinal flora, has been noted in children with autism [99,107] Dysbiosis can result from use of antibiotics. As beneficial bacteria are killed, antibiotic resistant pathogenic organisms can take their place. It has been theorized that toxins produced by pathogenic organisms may be affecting the brains of individuals with autism. In addition, decreased levels of disaccharide digestive enzymes have been noted in children with autism [99].

There are anecdotal reports of improvement of autistic behavior on restricted diets. Some experimental studies have reported improvements reported include socialization, speech, strange and unusual behavior [108,109], stereotyped behaviors, attention/hyperactivity [110] and physiological symptoms [109] One study of the casein/gluten free diet considered children with and without GI symptoms separately. They found greater improvent in autistic behaviors in children with gastrointestinal symptoms compared to those without [109]. The reported improvements may be due to several reasons. Removal of allergens may result in lessened autoimmune reactions [66]. Removal of gluten may reduce intestinal permeability [103,105]. Removal of dietary proteins for which there is insufficient enzymic activity may reduce dysbiosis [111].

The brain has the potential to directly effect the functioning of the gut. Stress has been implicated in Irritable Bowel Syndrome with alterations of the intestinal barrier function, altered balance in enteric microflora, exaggerated stress response and visceral hypersensitivity [112]. Antidepressants [113] and therapy [114] have been found to be effective treatments for irritable bowel syndrome (IBS) and inflammatory bowel disease (IBD). There is also a finding that the brains of patients with IBS have increased hypothalamic gray matter compared with controls, though it is unknown whether the brain changes result from long term IBS or are preexisting [115].

### Neurological system

Among the body systems involved in autism is obviously the brain. Anatomical differences in the cerebellum and amygdala have been noted in multiple studies, and other regions have been inconsistently identified as diverging from the average [116]. Decreases in Purkinje and granular cells have been noted [117]. Macrocephaly is present in about 20% of people with autism studied, with a general upward trend in brain size in other people with autism. The increase appears to be disproportionately from white matter enlargement. The cause of the macrocephaly is not known, though larger brains are prevalent among first degree, unaffected relatives. Neuroinflamation is one postulated cause [118].

Minicolumns in the neocortex have been postulated as the fundamental unit of cognition [119]. Minicolumns in autistic brains appear to be narrower, with tighter spacing and higher neuron density [120]. Whether this is a sign of pathology is unclear, as the same variation occurs in autopsies of three distinguished scientists [121]. Autism does occur more often in families or mathematicians, engineers and physicists [122]. It has been theorized that narrow minicolumns facilitate discrimination and more finely tuned activities, while wider minicolumns would facilitate generalization. This is consistent with the behavioral observations of stimulus overselectivity in autism. Stimulus overselectivity is the neglect of some features and the overly focused attention on other features, to the detriment of the observation of the whole [123]. Evidence also exists for an increased excitatory/inhibitory neuronal activity in the autistic brain [119,124].

Functional MRI studies are giving evidence to enhanced local connectivity, and reduced global connectivity in the autistic brain. This might result in an over analysis of smaller features and an impairment in synthesizing the information into a coherent whole [125]. It has been suggested that a feature in the development of autistic traits is a low signal to noise ratio in neural signals. In murine models, constant undifferentiated noise will indefinitely delay the maturation of neurons responsible for processing sound. A similar low signal to noise ratio in multiple systems in the autistic brain may be responsible for the impairments observed [126]. This would be consistent with the underconnectivity theory. Neuronal synchrony may be impaired if presynaptic and postsynaptic neurons don’t fire within ≪100 ms of each other [127].

Brain hypoperfusion has been noted in several studies of subjects with autism. Interestingly, the region affected can vary widely. Hypoperfusion can result from structural abnormalities or from global effects such as oxidative stress [7]. Seizures are present in 30% of people with autism [128]. In addition, subclinical seizures are often present and treatment with anti-epileptics can result in mental improvement [129,130].

### Modeling autism

**Figure 2:**
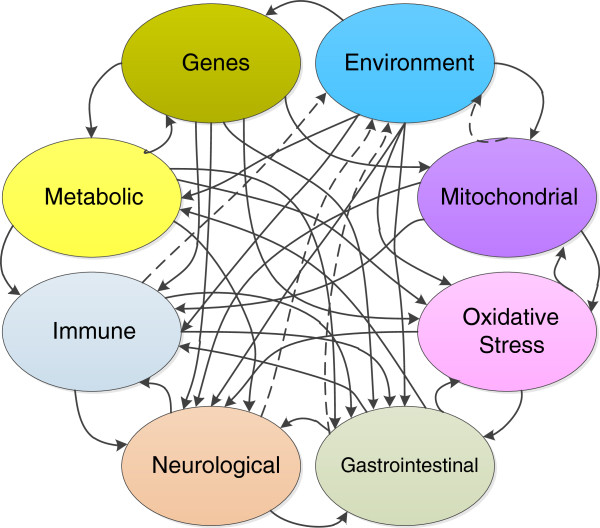
Proposed interactions between various elements implicated in autism.

All of the systems described above interact in highly complex ways. To date, little research exists in autism modeling outside of the genetic and neurological systems. Finding commonalities between autism and other conditions may lead to new treatments. Rzhetsky used statistical models to find genetic overlaps between autism, bipolar disorder and schizophrenia [131]. Individual subsystems of importance in autism have been modeled [132,133], but work needs to be done in modeling combinations of systems. It is clear that autism poses a challenging problem for modeling due to the high level of interactions between the different elements [134]. The probably incomplete Figure 2 shows some potential interactions between the systems discussed in this paper. For example, an analysis of children with both autism and mitochondrial disease found that a high proportion, 70%, regressed during a fever [135]. This illustrates just one example of an intersystem interaction between the mitochondrial, immune and neurological systems. 

The dotted lines in Figure 2 indicate how even the environment might be effected by the presence of autism. For example, food allergies or special diets would change the environment through different food choices. Fecal incontinence in older children would change the activities the child would be exposed to. Energy deficits from mitochondrial dysfunction could affect school activities. And being oversensitive to sensory input would change activities and family dynamics.

Much work has been done investigating the genetic basis of autism. Additional work needs to be done to find and cluster the genes involved in autism. Modeling autism will require an integration of both systems and scales. A few potential research areas are presented below.

### Hierarchical modeling

Modeling autism is complex due to the different physiological scales involved. Issues of importance to model range from the organ level to the genetic. Most systems biology to date has emphasized the “lower” levels, with a strong emphasis on direct genetic interactions. Outside of a few systems, such as the cardiovascular [136], less work has been done on an organ scale. To create a true model of the human body, the microscopic and macroscopic need to be integrated. One way to do this is to use a hierarchical system. Modules can be developed to model the scale being considered, with appropriate links between levels. Techniques have been borrowed from the systems engineering and software engineering communities to aid and formalize these connections between modules. An example is the BioUML, an open source platform for multilevel biology modeling [137]. Hierarchical modeling using rule based models has been implemented at a cellular level [138].

A hierarchical approach allows for separation of development of models for subsystems, but global effects of different substances and conditions need to be considered too. Studies of trans-organ and system effects of substances is a relatively unexplored field of study. For example, oxidative stress affects the mitochondria directly [24], but also the larger systems such as the brain [47]. Mitochondrial stress may also affect the brain indirectly. Dysregulation of mitochondrial dynamics has been implicated in Parkinson’s disease [139]. Mitochondrial stress may lead to lipid peroxidation leading to reactive aldehyde generation in the liver, and finally to microglial activation and neuronal death [61].

Inflammation can affect many body systems. Inflammation can also be part of a feedback mechanism where inflammation creates conditions which create or perpetuate inflammation [140]. Xenobiotic substances must be taken into account. Many exogenous substances are not typically included in existing models. Toxins such as PCBs, pesticides and heavy metals can affect the efficiency of enzymes often deficient in autism and need to be considered as a potential causative element [18,38]. In addition, the effect of toxins in combination may not be the same as the effect of the toxins in isolation [141-143]. The microbiome, the complex ecosystem of intestinal flora, may have an impact on many systems in the body either through immunological effects, or through the microbial metabolites such as the proprionic acid produced by clostridium [144]. Special diets and supplements used by many on the autism spectrum may affect the composition of the microbiome in addition to possibly changing the function of enzymes [145,146].

### Identifying subgroups

In spite of autism’s many common behaviors, it has become evident that autism has a complex etiology and multiple subgroups. The development of autism appears to be a complex interaction of genes and environmental factors. Since most cases of autism are idiopathic, there are an unknown number of subgroups that may be present. Treating autism as homogeneous will obscure the differences required to ascertain the variances needed for proper treatment. Identification of subgroups would aid in both research and treatments. As David Amaral, President of the International Society for Autism Research states, “There is not going to be rapid progress in autism research unless we subtype” [147]. This subtyping can be done on the basis of genes or clinical data. Clustering has been tried using behavioral symptoms but has had little success at identifying latent factors [148-150].

The benefits of subgrouping are as follows. Subgrouping the population might result in subgroups that have distinctive symptoms and pathology that are already familiar in the medical literature, and can draw upon treatments that work in existing treatable conditions. For example, if one subgroup is a variant of a known syndrome, we can possibly benefit from the treatments known in the context of that syndrome. Subtyping would reduce the use of therapeutic trials, allowing a more targeted treatment. Another benefit that accrues from subgrouping is in prevention. If we know the sequelae of another similar condition, we can take appropriate action to include appropriate preventive measures in the treatment protocol. For example, if seizures are a symptom of the similar known syndrome or condition, potentially a periodic EEG evaluation could be included in the treatment protocol.

Biomarkers can be used for clustering subgroups. Many of the metabolic, immunologic, proteomic, genetic and anatomical differences listed above can be used to search for subgroups [151,152]. Biomarkers can also be identified with more advanced methods [16,153]. An important consideration is that the biomarkers used be clinically relevant, chosen to maximize the potential for treatment [154]. For example, the following parameters could be included in a feature vector in the subgroup calculation algorithm, for the purpose of clustering:

 – Genetics. This can include genetic panels such as mitochondrial or results of microarray testing.

 – Lab test results, such as the above mentioned metabolic, immunologic and proteomic biomarkers.

 – Symptoms and severity as a function of time. These could be “hard” symptoms such as the presence and type of epilepsy, or “soft” symptoms such as parent reports of sociability.

 – Treatments and their effectiveness. The treatments could include steps to address some of the disease markers discussed above, such as methycobalamin and folinic acid [36] for methylation issues and carnitine [61] for mitochondrial issues.

The feature vector would be a vector with both specific values and binary numbers as markers such as a 1 for the presence of a polymorphism or other hard symptom and a 0 for none. For example, a child with the MTHFR 677 genotype, a tGSH: GSSG ratio of 8.6 and no epilepsy could be represented by the feature vector [1, 8.6, 0]. Once in numerical form, a variety of pattern recognition techniques can be used.

One popular clustering technique is the K-means [155]. The k-means algorithm is essentially a density finder. It assigns each input vector using an indicator function to a cluster defined by a prototype vector. The algorithm then minimizes the global average squared Euclidean distance from each input vector to the prototype. This optimization changes the position of the prototype vector to reflect Euclidean density patterns. The prototype center is the average of the input vectors assigned to it and thus potentially representative of a subgroup.

One weakness of the k-means is that it performs a hard assignment of each input vector to a cluster. An input vector is either entirely in a cluster or not at all. This would not match situations where there might be an overlap of symptoms. Fuzzy techniques would be of value in these cases. Fuzzy set theory allows intermediate levels, between 0 and 1, of belonging to member sets. The fuzzy c-means (FCM) is a fuzzy generalization of the k-means algorithm to allow input vectors to belong to more than one prototype [156]. The FCM also does not suffer from the stability problems that sometimes occur in the k-means when an input vector will switch back and forth between two prototypes, and thus changing the prototypes in the process.

An important issue with clustering algorithms is the number and validity of clusters. The k-means and FCM algorithms will find the number of clusters specified during program initialization, regardless of the actual number of clusters. Some clustering algorithms can produce clusters that are empty or degenerate. Many practitioners will heuristically try different numbers of clusters and asses the fit. There are also various methods to attempt to quantify the validity of clusters [157]. The Self Organizing Map (SOM) maintains a proximity relationship between clusters and can be useful for visualization [158].

The above techniques are *unsupervised*. Unsupervised techniques relay wholly on the input data to find clusters or groupings in the data. Supervised techniques incorporate additional knowledge about the expected groupings to guide the cluster development process. This additional information, if available, can aid in complex and high dimensional problems. Support Vector Machines [159,160] and a variety of Neural Network algorithms can be used to find patterns in the data [161]. Although supervised algorithms can, in general, outperform unsupervised algorithms, additional “ground truth” data is often unavailable. This ground truth can be information such as genes already associated with a phenotype or reaction to an intervention. It could also be symptoms that could also be used as inputs, such as the before mentioned presence of epilepsy.

Most of the algorithms mentioned above measure similarity based on the Euclidean distance metric. Euclidean and other Minkowski lp norms such as the “city block” distance measures will represent hyperspherical patterns well. Other distance measures are possible such as various correlation measures [162] and non-spherical distance measures such as the Mahalanobis distance [161]. Another issue is the scale of the data. Expected results in lab tests may vary by several orders of magnitude. Therefore, it is usually advisable to normalize the data before using in an algorithm.

Perhaps the most critical issue is the “Curse of Dimensionality”. The curse of dimensionality refers to the somewhat counterintuitive properties of high dimensional spaces whereby additional information can result in a lessening of discernment. The simplest of the implications of high dimensional space is that the amount of data required to adequately cover a volume increases exponentially with dimension. It can be shown geometrically that most of the volume of a high dimensional Gaussian is contained in its tails, rather than at its center. This has obvious implications to distance based algorithms. The distance from a center of a cluster to any point is concentrated in a small interval and the relative differences from various data points to the prototype become essentially the same. Thus discriminatory power can decrease with added information, even if that additional information has discriminatory power in of itself [163,164]. That has implications for finding subgroups in a complicated disease such as autism that might require a large number of features. Feature selection will alleviate the curse of dimensionality but may exclude features needed to find less prevalent subgroups. The curse of dimensionality may also be avoided by using subspace methods or hierarchical clustering.

Another issue prevalent in autism data is the abundance of missing data. One cause of missing data would be different protocols for different studies resulting in similar but not identical feature vectors. When utilizing clinical data, physicians will not perform all tests on all patients, resulting in missing data when patients are combined. Therefore techniques need to be utilized to make the most of the data that is present [165,166].

Numerical data in autism research has particular challenges. The data can refer to disparate body systems. Data can be problematic to integrate across studies and research centers. For example, studies can have different selection criteria, experimental conditions, and goals. Research centers can have different testing procedures which can lead to varying results. Data is often not precise. Fuzzy techniques should be incorporated, as many of the data considered will not be easily quantifiable, such as parent reports of behavior. Also, what might be considered outlier data may in fact be important. It may be representative of the extreme values that are evident in autism data [167]. There are a myriad of information that might be useful in determining autism phenotypes. As mentioned before, it might include items such as genotype information and lab results. It also might include items such as parent ratings of diarrhea odor. It is obvious that a value of ‘1’ in these three categories would have very different meanings, although numerically they would be the same.

Incorporating domain knowledge into the identification of subgroups will alleviate many of the problems noted above. As shown in the preceding sections, there is much qualitative information about autism contained in the medical literature. Most of it is single system studies. Techniques need to be discovered to integrate this information together. One way to incorporate domain knowledge is to embed causal information into the solution [168].

Some preliminary, simple subgrouping has already shown promise. An analysis of the gluten and casein elimination diet showed greater improvement in symptoms in children with gastrointestinal symptoms compared to those without [109]. This information can help practitioners decide whether to recommend restrictive diets. It has been proposed that there may be a mitochondrial [58], intestinal permeability [103] and immune subgroups [11] in autism, but it is probably more complex than that as many children may belong to multiple subgroups. Thus it is imperative to develop subgroups that have clinical significance for treating the symptoms of autism, not just statistical validity.

For example, one could, possibly discover a subtype of autism that presents with clinical or subclinical seizures of a certain characteristic type. The treatment of seizures being a well-studied area, by itself, we could potentially establish a treatment protocol for patients in this subgroup, using treatment studies of drugs used for seizures in these patients also presenting with autism. This would result in a new treatment for those with autism, in contrast to using a seizure medication as an off-label drug without clear evidence of efficacy in this population.

### Time dependent modeling

Another issue of importance is the time scales involved. Autism is a developmental, not a static disease. Disease progression might start prenatally and extend throughout childhood. And of course, the child’s body is growing and changing. Modeling incorporating time progression has been primarily on the genetic or cellular level. Frameworks have been developed for parameter adjustments during phenotype transitions [169]. Molecular connectivity maps incorporating differentially expressed genes have been used to investigate the relationship of aging to neurological and psychiatric diseases [170].

Another time range to be considered is the progression through generations. Transgenerational changes have been shown with common toxicants. Low level bisphenol A exposure during pregnancy in mice resulted in transgenerational alterations in gene expression and behavior [171]. Another possible avenue for children with autism would be that impairments in the mother’s methylation and sulfur pathways might result in a concentration of toxins in a mother. She would then pass on a greater than normal amount of toxins to her child prenatally [172] and through breast feeding [173]. This will impair the detoxification systems of the child from an early age, resulting in an even greater build-up of toxins. If this child, a girl, has children, she would pass on an even greater toxic load to her children. As the effects of toxins are more severe the earlier they are introduced, this might lead to developmental delays, including autism. Thus a non-genetic, non-epigenetic trans-generational inheritance could be occurring. A recent study showed a three-fold rate increase of autism in the descendants of survivors of the mercury induced Pink disease (infantile acrodynia). The study did not separate out matrilineal descendants, so it is impossible to determine whether there were toxins passed in utero, or whether the increased incidence was a result of a genetic hypersensitivity to mercury [174]. This sort of inheritance can also happen in other systems [175]. Inducement of diabetes in pregnant rats will result in increased prevalence of diabetes and obesity in the offspring. This can lead to gestational diabetes in the children and perpetuation of the diabetes through generations, through environmental causes [176,177].

Another source of time-dependence is that the brain itself is a state machine, in the sense that future characteristics depend on past characteristics, the various interventions employed or not employed at a certain time, etc. Simplified modeling with reasonable assumptions can be potentially employed to answer questions of generic value. An example would be “Are outcomes better for children with regression, who were treated with anti-epileptic medications prior to puberty, compared to children who received such treatment later, after puberty?” Another example would be “Do children who exhibit conditions such as gastro-intestinal abnormalities or seizures generally tend to lose these symptoms after a certain age?” If so, did these children receive a certain therapy, either medical, educational or behavioral, at a certain age? In summary, a time-dependent model will throw more light into brain plasticity and its contribution to the outcomes that we see in this population.

In order to introduce this complexity, we propose enhancing our models using Dynamic Time Warping (DTW) [178] or a more complex model with state information, similar to hidden Markov models where the body is assumed to be in a state where it produces certain symptoms or observations and transitions to other states based on the model. Estimating these models and predicting outcomes would be the most complex of the techniques proposed in this article, and would be the goal for modeling such a complex time-varying system.

## Discussion and conclusions

This paper contains, of necessity, an incomplete review of the issues involved in autism. Research is exploding in this area and new findings are being published every month.

It is clear that the complexity of autism presents a both challenge and an opportunity for systems biologists. Modeling autism requires new techniques to be developed to harness and tame the complexity of interactions. For example, a possible interaction would be impairment of the detoxification system could allow toxins to accumulate and cause mitochondrial dysfunction [48,49], which could cause immune dysfunction [179,180], which could cause gastrointestinal dysfunction which could then affect the brain [181]. This is not to imply that this relation is the cause of autism. In fact, the whole relation could go the other way, with stress inducing bowel dysfunction [112]. The bowel dysfunction could, through opening of tight junctions, induce immune activation [182], which could contribute to mitochondrial dysfunction [72], and finally the resultant oxidative stress can cause more resources to be used in the production of GSH, perturbing the metabolic pathways [183]. And in fact, the chain is not ordered. Gastrointestinal dysfunction could impair mitochondrial function directly through the clostridial production of proprionic acid [8]. These interactions are a purely hypothetical thought experiment and are not to be represented as causes. But even so, it is apparent that the number of possible interactions of systems in autism is almost exponential. This necessitates a system approach.

Autism could be considered a model for other complex diseases. The probable interplay between genetic and environmental factors is suspected as a factor in many diseases such as cancer and diabetes. Since many of the genetic variants that predispose children to autism are common in the general population, findings in autism may have much broader implications for the population in general.

Autism is the most rapidly increasing developmental disability with enormous costs to individuals and to society. The importance of modeling autism research cannot be overstated. In summary, the goal of a systems approach to modeling autism, can potentially lead to the following concrete benefits. First, having a comprehensive evolving digital data model for autism gives us a platform to capture the on-going research in an analysable format. The model itself can “learn” as results are incorporated as training data, into the system. Second, immediate tools such as a detailed hierarchical Intake or Follow up questionnaire could result from the system, based on its knowledge of subtypes and interconnections, leading to better clinical care for this population. Third, the system can be used for hypothesis generation, suggesting possible research topics for clinical trials.

Autism research findings need to be mined, integrated and modeled to help not just future generations, but also to improve the outcomes for the current generation of people with autism.

## Competing Interests

The authors declare that they have no competing interests.

## Authors’ contributions

MR wrote the document with written and theoretical contributions by PS. Both authors reviewed and approved the document.

## Authors’ information

MR is an assistant professor of Systems Engineering and Computer Engineering at the University of Houston – Clear Lake. PS is the CEO of MedicalMine Inc, a company involved in developing technological tools for improving clinical care and research in autism and other chronic conditions.

## References

[B1] Fombonne E Epidemiological surveys of autism and other pervasive developmental disorders: an update J Autism Dev Disord 2003 33 365 382 12959416 10.1023/a:1025054610557

[B2] Baio J Prevalence of Autism Spectrum Disorders — Autism and Developmental Disabilities Monitoring Network, 14 Sites, United States, 2008 Book Prevalence of Autism Spectrum Disorders Autism and Developmental Disabilities Monitoring Network 14 Sites, United States, 2008 2012 City: Surveillance Summaries 1 19 22456193

[B3] Association AP Diagnostic and statistical manual of mental disorders Book Diagnostic and statistical manual of mental disorders 1994 4 City: City

[B4] Ganz ML The Lifetime Distribution of the Incremental Societal Costs of Autism Arch Pediatr Adolesc Med 2007 161 343 349 17404130 10.1001/archpedi.161.4.343

[B5] Severson K Aune J Jodlowski D Osteen M Bruno Bettelheim, Autism, and the Rhetoric of Scientific Authority Autism and representation 2008 New York: Routledge Routledge research in cultural and media studies

[B6] Wegiel J Kuchna I Nowicki K Imaki H Wegiel J Marchi E Ma S Chauhan A Chauhan V Bobrowicz T The neuropathology of autism: defects of neurogenesis and neuronal migration, and dysplastic changes Acta Neuropathol 2010 119 755 770 20198484 10.1007/s00401-010-0655-4 PMC2869041

[B7] Herbert M Autism: A brain disorder or a disorder that affects the brain? Neurotoxicology 2006 27 1151 1152

[B8] Rossignol DA Bradstreet JJ Evidence of Mitochondrial Dysfunction in Autism and Implications for Treatment Am J Biochem Biotechnol 2008 4 208 217

[B9] Tsiouris J Kim S-Y Brown W Pettinger J Cohen I Prevalence of Psychotropic Drug Use in Adults with Intellectual Disability: Positive and Negative Findings from a Large Scale Study J Autism Dev Disord 2012 1 13 22829245 10.1007/s10803-012-1617-6

[B10] Buie T Campbell DB Fuchs GJ III Furuta GT Levy J VandeWater J Whitaker AH Atkins D Bauman ML Beaudet AL Evaluation, Diagnosis, and Treatment of Gastrointestinal Disorders in Individuals With ASDs: A Consensus Report Pediatrics 2010 125 S1 S18 20048083 10.1542/peds.2009-1878C

[B11] Ashwood P Wills S Van de Water J The immune response in autism: a new frontier for autism research J Leukoc Biol 2006 80 1 15 16698940 10.1189/jlb.1205707

[B12] Hansen RL Ozonoff S Krakowiak P Angkustsiri K Jones C Deprey LJ Le D-N Croen LA Hertz-Picciotto I Regression in Autism: Prevalence and Associated Factors in the CHARGE Study Ambul Pediatr 2008 8 25 31 18191778 10.1016/j.ambp.2007.08.006

[B13] Folstein SE Rosen-Sheidley B Genetics of autism: complex aetiology for a heterogeneous disorder Nat Rev Genet 2001 2 943 955 11733747 10.1038/35103559

[B14] Freitag CM The genetics of autistic disorders and its clinical relevance: a review of the literature Mol Psychiatry 2007 12 2 22 17033636 10.1038/sj.mp.4001896

[B15] James SJ Zimmerman AW Oxidative Stress and the Metabolic Pathology of Autism Autism Current Theories and Evidence 2008 NJ USA: Humana Press, Totowa 245 268

[B16] Gohlke J Thomas R Zhang Y Rosenstein M Davis A Murphy C Becker K Mattingly C Portier C Genetic and environmental pathways to complex diseases BMC Syst Biol 2009 3 46 19416532 10.1186/1752-0509-3-46 PMC2680807

[B17] Herbert MR Russo JP Yang S Roohi J Blaxill M Kahler SG Cremer L Hatchwell E Autism and environmental genomics 2006 27 671 684 10.1016/j.neuro.2006.03.017 16644012

[B18] Deth R Muratore C Benzecry J Power-Charnitsky V-A Waly M How environmental and genetic factors combine to cause autism: A redox/methylation hypothesis 2008 29 190 201 10.1016/j.neuro.2007.09.010 18031821

[B19] Campbell DB Buie TM Winter H Bauman M Sutcliffe JS Perrin JM Levitt P Distinct genetic risk based on association of MET in families with co-occurring autism and gastrointestinal conditions Pediatrics 2009 123 1018 1024 19255034 10.1542/peds.2008-0819

[B20] Campbell DB D'Oronzio R Garbett K Ebert PJ Mirnics K Levitt P Persico AM Disruption of cerebral cortex MET signaling in autism spectrum disorder Ann Neurol 2007 62 243 250 17696172 10.1002/ana.21180

[B21] James SJ Zimmerman AW Oxidative Stress and the Metabolic Pathology of Autism Autism: current theories and evidence 2008 NJ: Humana Press, Totowa xix 474 p

[B22] Deth R Muratore C Chauhan A, Chauhan V The Redox/Methylation Hypothosis of Autism: A Molecular Mechanism for Heavy Metal-Induced Neurotoxicity Autism: Oxidative Stress, Inflammation, and Immune Abnormalities 2010 FL USA: CRC Press, Boca Raton

[B23] Schanen NC Epigenetics of autism spectrum disorders Hum Mol Genet 2006 15 2 R138 R150 16987877 10.1093/hmg/ddl213

[B24] Ott M Gogvadze V Orrenius S Zhivotovsky B Mitochondria, oxidative stress and cell death Apoptosis 2007 12 913 922 17453160 10.1007/s10495-007-0756-2

[B25] James SJ Zimmerman AW Oxidative Stress and the Metabolic Pathology of Autism Autism Current Theories and Evidence Current Clinical Neurology, Volume 2009 AK USA: Humana Press, Little Rock 245 268

[B26] James SJ Cutler P Melnyk S Jernigan S Janak L Gaylor DW Neubrander JA Metabolic biomarkers of increased oxidative stress and impaired methylation capacity in children with autism Am J Clin Nutr 2004 80 1611 1617 15585776 10.1093/ajcn/80.6.1611

[B27] James SJ Melnyk S Jernigan S Cleves MA Halsted CH Wong DH Cutler P Bock K Boris M Bradstreet JJ Metabolic endophenotype and related genotypes are associated with oxidative stress in children with autism Am J Med Genet B Neuropsychiatr Genet 2006 141B 947 956 16917939 10.1002/ajmg.b.30366 PMC2610366

[B28] Yao Y Walsh WJ McGinnis WR Pratico D Altered vascular phenotype in autism: correlation with oxidative stress Arch Neurol 2006 63 1161 1164 16908745 10.1001/archneur.63.8.1161

[B29] Loeken MR Free radicals and birth defects J Matern Fetal Neonatal Med 2004 15 6 14 15101606 10.1080/14767050310001650662

[B30] Dringen R Glutathione metabolism and oxidative stress in neurodegeneration Eur J Biochem 2000 267 4903 10931171 10.1046/j.1432-1327.2000.01651.x

[B31] Kovacic P Jacintho JD Systemic lupus erythematosus and other autoimmune diseases from endogenous and exogenous agents: unifying theme of oxidative stress Mini Rev Med Chem 2003 3 568 575 12871159 10.2174/1389557033487926

[B32] Yao JK Leonard S Reddy R Altered glutathione redox state in schizophrenia Dis Markers 2006 22 83 93 16410648 10.1155/2006/248387 PMC3850558

[B33] Frey BN Andreazza AC Kunz M Gomes FA Quevedo J Salvador M Goncalves CA Kapczinski F Increased oxidative stress and DNA damage in bipolar disorder: a twin-case report Prog Neuropsychopharmacol Biol Psychiatry 2007 31 283 285 16859818 10.1016/j.pnpbp.2006.06.011

[B34] Tilleux S Hermans E Neuroinflammation and regulation of glial glutamate uptake in neurological disorders J Neurosci Res 2007 85 2059 2070 17497670 10.1002/jnr.21325

[B35] Yao JK Reddy RD van Kammen DP Oxidative damage and schizophrenia: an overview of the evidence and its therapeutic implications CNS Drugs 2001 15 287 310 11463134 10.2165/00023210-200115040-00004

[B36] James SJ Melnyk S Fuchs G Reid T Jernigan S Pavliv O Hubanks A Gaylor DW Efficacy of methylcobalamin and folinic acid treatment on glutathione redox status in children with autism Am J Clin Nutr 2009 89 425 430 19056591 10.3945/ajcn.2008.26615 PMC2647708

[B37] Hertz-Picciotto I Croen LA Hansen R Jones CR van de Water J Pessah IN The CHARGE study: an epidemiologic investigation of genetic and environmental factors contributing to autism Environ Health Perspect 2006 114 1119 1125 16835068 10.1289/ehp.8483 PMC1513329

[B38] Pessah IN Seegal RF Lein PJ LaSalle J Yee BK Van De Water J Berman RF Immunologic and neurodevelopmental susceptibilities of autism 2008 29 532 545 10.1016/j.neuro.2008.02.006 PMC2475601 18394707

[B39] Andreyev AI Kushnareva YE Starkov AA Mitochondrial metabolism of reactive oxygen species Biochem Moscow 2005 70 200 214 10.1007/s10541-005-0102-7 15807660

[B40] Skladal D Sudmeier C Konstantopoulou V Stöckler-Ipsiroglu S Plecko-Startinig B Bernert G Zeman J Sperl W The Clinical Spectrum of Mitochondrial Disease in 75 Pediatric Patients Clin Pediatr 2003 42 703 710 10.1177/000992280304200806 14601919

[B41] Rossignol DA Frye RE Mitochondrial dysfunction in autism spectrum disorders: a systematic review and meta-analysis Mol Psychiatry 2011 17 3 290 314 21263444 10.1038/mp.2010.136 PMC3285768

[B42] Weissman JR Kelley RI Bauman ML Cohen BH Murray KF Mitchell RL Kern RL Natowicz MR Mitochondrial disease in autism spectrum disorder patients: a cohort analysis PLoS One 2008 3 e3815 19043581 10.1371/journal.pone.0003815 PMC2584230

[B43] Ramoz N Reichert JG Smith CJ Silverman JM Bespalova IN Davis KL Buxbaum JD Linkage and association of the mitochondrial aspartate/glutamate carrier SLC25A12 gene with autism Am J Psychiatr 2004 161 662 669 15056512 10.1176/appi.ajp.161.4.662

[B44] Graf WD Marin-Garcia J Gao HG Pizzo S Naviaux RK Markusic D Barshop BA Courchesne E Haas RH Autism associated with the mitochondrial DNA G8363A transfer RNA(Lys) mutation J Child Neurol 2000 15 357 361 10868777 10.1177/088307380001500601

[B45] Filipek PA Juranek J Smith M Mays LZ Ramos ER Bocian M Masser-Frye D Laulhere TM Modahl C Spence MA Gargus JJ Mitochondrial dysfunction in autistic patients with 15q inverted duplication Ann Neurol 2003 53 801 804 12783428 10.1002/ana.10596

[B46] Silverman JM Buxbaum JD Ramoz N Schmeidler J Reichenberg A Hollander E Angelo G Smith CJ Kryzak LA Autism-related routines and rituals associated with a mitochondrial aspartate/glutamate carrier SLC25A12 polymorphism Am J Med Genet B 2008 147B 408 410 10.1002/ajmg.b.30614 17894412

[B47] Lin MT Beal MF Mitochondrial dysfunction and oxidative stress in neurodegenerative diseases Nature 2006 443 787 795 17051205 10.1038/nature05292

[B48] Mori N Yasutake A Marumoto M Hirayama K Methylmercury inhibits electron transport chain activity and induces cytochrome ≪ i > c≪/i > release in cerebellum mitochondria J Toxicol Sci 2011 36 253 259 21628953 10.2131/jts.36.253

[B49] Tonazzi A Indiveri C Effects of heavy metal cations on the mitochondrial ornithine/citrulline transporter reconstituted in liposomes Biometals 2011 24 1205 1215 21769608 10.1007/s10534-011-9479-5

[B50] Karami-Mohajeri S Abdollahi M Toxic influence of organophosphate, carbamate, and organochlorine pesticides on cellular metabolism of lipids, proteins, and carbohydrates Hum Exp Toxicol 2011 30 1119 1140 21071550 10.1177/0960327110388959

[B51] Correia C Coutinho AM Diogo L Grazina M Marques C Miguel T Ataide A Almeida J Borges L Oliveira C Brief report: High frequency of biochemical markers for mitochondrial dysfunction in autism: no association with the mitochondrial aspartate/glutamate carrier SLC25A12 gene J Autism Dev Disord 2006 36 1137 1140 17151801 10.1007/s10803-006-0138-6

[B52] Gargus JJ Faiqa I Mitochondrial Energy-Deficient Endophenotype in Autism Book Mitochondrial Energy-Deficient Endophenotype in Autism 2008 City: Science Publications

[B53] Oliveira G Diogo L Grazina M Garcia P Ataide A Marques C Miguel T Borges L Vicente AM Oliveira CR Mitochondrial dysfunction in autism spectrum disorders: a population-based study Dev Med Child Neurol 2005 47 185 189 15739723 10.1017/s0012162205000332

[B54] Russo AJ Decreased Serum Cu/Zn SOD in Children with Autism Nutr Metabol Insights 2010 2009 27

[B55] Gargus J Chauhan A, Chauhan V Mitochondrial Component of Calcium Signaling Abnormality in Autism Autism, Oxidative Stress, Inflamation and Immune Abnormalities 2010 Boca Raton FL USA: CRC Press 207 224

[B56] Palmieri L Papaleo V Porcelli V Scarcia P Gaita L Sacco R Hager J Rousseau F Curatolo P Manzi B Altered calcium homeostasis in autism-spectrum disorders: evidence from biochemical and genetic studies of the mitochondrial aspartate/glutamate carrier AGC1 Mol Psychiatry 2010 15 38 52 18607376 10.1038/mp.2008.63

[B57] Fatemi SH Aldinger KA Ashwood P Bauman ML Blaha CD Blatt GJ Chauhan A Consensus Paper: Pathological role of the cerebellum in autism The Cerebellum 2012 1 31 22370873 10.1007/s12311-012-0355-9 PMC3677555

[B58] Frye RE Rossignol DA Mitochondrial Dysfunction Can Connect the Diverse Medical Symptoms Associated With Autism Spectrum Disorders Pediatr Res 2011 69 41R 47R 10.1203/PDR.0b013e318212f16b PMC3179978 21289536

[B59] Arpaia E Benveniste P Di Cristofano A Gu Y Dalal I Kelly S Hershfield M Pandolfi PP Roifman CM Cohen A Mitochondrial Basis for Immune Deficiency J Exp Med 2000 191 2197 2208 10859343 10.1084/jem.191.12.2197 PMC2193200

[B60] Hao J Shen W Tian C Liu Z Ren J Luo C Long J Sharman E Liu J Mitochondrial nutrients improve immune dysfunction in the type 2 diabetic Goto-Kakizaki rats J Cell Mol Med 2009 13 701 711 18410524 10.1111/j.1582-4934.2008.00342.x PMC3822877

[B61] Pastural É Ritchie S Lu Y Jin W Kavianpour A Khine Su-Myat K Heath D Wood PL Fisk M Goodenowe DB Novel plasma phospholipid biomarkers of autism: Mitochondrial dysfunction as a putative causative mechanism Prostaglandins Leukot Essent Fatty Acids 2009 81 253 264 19608392 10.1016/j.plefa.2009.06.003

[B62] Sweeten TL Bowyer SL Posey DJ Halberstadt GM McDougle CJ Increased prevalence of familial autoimmunity in probands with pervasive developmental disorders Pediatrics 2003 112 e420 14595086 10.1542/peds.112.5.e420

[B63] Ashwood P Anthony A Torrente F Wakefield AJ Spontaneous mucosal lymphocyte cytokine profiles in children with autism and gastrointestinal symptoms: mucosal immune activation and reduced counter regulatory interleukin J Clin Immunol 2004 10 664 673 15622451 10.1007/s10875-004-6241-6

[B64] Gupta S Aggarwal S Rashanravan B Lee T Th1- and Th2-like cytokines in CD4+ and CD8+ T cells in autism J Neuroimmunol 1998 85 106 109 9627004 10.1016/s0165-5728(98)00021-6

[B65] Molloy CA Morrow AL Meinzen-Derr J Schleifer K Dienger K Manning-Courtney P Altaye M Wills-Karp M Elevated cytokine levels in children with autism spectrum disorder J Neuroimmunol 2006 172 198 205 16360218 10.1016/j.jneuroim.2005.11.007

[B66] Jyonouchi H Autism spectrum disorders and allergy: observation from a pediatric allergy/immunology clinic Expert Rev Clin Immunol 2010 6 397 411 20441426 10.1586/eci.10.18

[B67] Croonenberghs J Wauters A Devreese K Verkerk R Scharpe S Bosmans E Egyed B Deboutte D Maes M Increased serum albumin, gamma globulin, immunoglobulin IgG, and IgG2 and IgG4 in autism Psychol Med 2002 32 1457 1463 12455944 10.1017/s0033291702006037

[B68] Furlano RI Anthony A Day R Brown A McGarvey L Thomson MA Davies SE Berelowitz M Forbes A Wakefield AJ Colonic CD8 and gamma delta T-cell infiltration with epithelial damage in children with autism J Pediatr 2001 138 366 372 11241044 10.1067/mpd.2001.111323

[B69] Trajkovski V Ajdinski L Spiroski M Plasma concentration of immunoglobulin classes and subclasses in children with autism in the Republic of Macedonia: retrospective study Croat Med J 2004 45 746 749 15578810

[B70] Krause I He XS Gershwin ME Shoenfeld Y Brief report: immune factors in autism: a critical review J Autism Dev Disord 2002 32 337 345 12199139 10.1023/a:1016391121003

[B71] Vargas DL Nascimbene C Krishnan C Zimmerman AW Pardo CA Neuroglial activation and neuroinflammation in the brain of patients with autism Ann Neurol 2005 57 67 81 15546155 10.1002/ana.20315

[B72] Palmieri L Persico AM Mitochondrial dysfunction in autism spectrum disorders: Cause or effect? Biochim Biophys Acta (BBA) Bioenerg 2010 1797 1130 1137 10.1016/j.bbabio.2010.04.018 20441769

[B73] Zhang B Angelidou A Alysandratos K-D Vasiadi M Francis K Asadi S Theoharides A Sideri K Lykouras L Kalogeromitros D Theoharides T Mitochondrial DNA and anti-mitochondrial antibodies in serum of autistic children J Neuroinflammation 2010 7 80 21083929 10.1186/1742-2094-7-80 PMC3001695

[B74] Pardo-VIllamizar C Zimmerman A Abha Chauhan VC Inflamation and Neuroimmunity in the Pathogenisis of Autism Autism: Oxidative Stress, Inflammation, and Immune Abnormalities 2009 Boca Raton FL USA: CRC Press 225 244

[B75] Doorduin J de Vries EFJ Willemsen ATM de Groot JC Dierckx RA Klein HC Neuroinflammation in Schizophrenia-Related Psychosis: A PET Study J Nucl Med 2009 50 1801 1807 19837763 10.2967/jnumed.109.066647

[B76] Rao JS Harry GJ Rapoport SI Kim HW Increased excitotoxicity and neuroinflammatory markers in postmortem frontal cortex from bipolar disorder patients Mol Psychiatry 2010 15 384 392 19488045 10.1038/mp.2009.47 PMC2844920

[B77] Eikelenboom P Bate C Van Gool WA Hoozemans JJM Rozemuller JM Veerhuis R Williams A Neuroinflammation in Alzheimer's disease and prion disease Glia 2002 40 232 239 12379910 10.1002/glia.10146

[B78] Wager-Smith K Markou A Depression: A repair response to stress-induced neuronal microdamage that can grade into a chronic neuroinflammatory condition? Neurosci Biobehav Rev 2011 35 742 764 20883718 10.1016/j.neubiorev.2010.09.010 PMC3777427

[B79] Tsuchiya KJ Hashimoto K Iwata Y Tsujii M Sekine Y Sugihara G Matsuzaki H Suda S Kawai M Nakamura K Decreased Serum Levels of Platelet-Endothelial Adhesion Molecule (PECAM-1) in Subjects with High-Functioning Autism: A Negative Correlation with Head Circumference at Birth Biol Psychiatry 2007 62 1056 1058 17509538 10.1016/j.biopsych.2006.12.018

[B80] Ashwood P Enstrom A Krakowiak P Hertz-Picciotto I Hansen RL Croen LA Ozonoff S Pessah IN de Water JV Decreased transforming growth factor beta1 in autism: A potential link between immune dysregulation and impairment in clinical behavioral outcomes J Neuroimmunol 2008 204 149 153 18762342 10.1016/j.jneuroim.2008.07.006 PMC2615583

[B81] Grigorenko EL Han SS Yrigollen CM Leng L Mizue Y Anderson GM Mulder EJ de Bildt A Minderaa RB Volkmar FR Macrophage Migration Inhibitory Factor and Autism Spectrum Disorders Pediatrics 2008 122 e438 e445 18676531 10.1542/peds.2007-3604 PMC3816765

[B82] Onore C Enstrom A Krakowiak P Hertz-Picciotto I Hansen R de Water JV Ashwood P Decreased cellular IL-23 but not IL-17 production in children with autism spectrum disorders J Neuroimmunol 2009 216 126 129 19800697 10.1016/j.jneuroim.2009.09.005 PMC2981175

[B83] Enstrom A Krakowiak P Onore C Pessah IN Hertz-Picciotto I Hansen RL Van de Water JA Ashwood P Increased IgG4 levels in children with autism disorder Brain Behav Immun 2009 23 389 395 19136055 10.1016/j.bbi.2008.12.005 PMC2696343

[B84] Enstrom AM Onore CE Van de Water JA Ashwood P Differential monocyte responses to TLR ligands in children with autism spectrum disorders Brain Behav Immun 2010 24 64 71 19666104 10.1016/j.bbi.2009.08.001 PMC3014091

[B85] Ashwood P Krakowiak P Hertz-Picciotto I Hansen R Pessah I Van de Water J Elevated plasma cytokines in autism spectrum disorders provide evidence of immune dysfunction and are associated with impaired behavioral outcome Brain Behav Immun 2011 25 40 45 20705131 10.1016/j.bbi.2010.08.003 PMC2991432

[B86] Boris M Goldblatt A Pollen Exposure as a Cause for the Deterioration of Neurobehavioral Function in Children with Autism and Attention Deficit Hyperactive Disorder: Nasal Pollen Challenge J Nutr Environ Med 2004 14 47 54

[B87] Curran LK Newschaffer CJ Lee L-C Crawford SO Johnston MV Zimmerman AW Behaviors Associated With Fever in Children With Autism Spectrum Disorders Pediatrics 2007 120 e1386 e1392 18055656 10.1542/peds.2007-0360

[B88] Mehler MF Purpura DP Autism, fever, epigenetics and the locus coeruleus Brain Res Rev 2009 59 388 392 19059284 10.1016/j.brainresrev.2008.11.001 PMC2668953

[B89] Kipnis J Cohen H Cardon M Ziv Y Schwartz M T cell deficiency leads to cognitive dysfunction: implications for therapeutic vaccination for schizophrenia and other psychiatric conditions Proc Natl Acad Sci USA 2004 101 8180 8185 15141078 10.1073/pnas.0402268101 PMC419577

[B90] Frye RE Sequeira JM Quadros EV James SJ Rossignol DA Cerebral folate receptor autoantibodies in autism spectrum disorder Mol Psychiatry 2012 10.1038/mp.2011.175 PMC3578948 22230883

[B91] Ramaekers VT Rothenberg SP Sequeira JM Opladen T Blau N Quadros EV Selhub J Autoantibodies to Folate Receptors in the Cerebral Folate Deficiency Syndrome N Engl J Med 2005 352 1985 1991 15888699 10.1056/NEJMoa043160

[B92] Ramaekers VT Sequeira JM Blau N Quadros EV A milk-free diet downregulates folate receptor autoimmunity in cerebral folate deficiency syndrome Dev Med Child Neurol 2008 50 346 352 18355335 10.1111/j.1469-8749.2008.02053.x PMC2715943

[B93] Gupta S Aggarwal S Heads C Brief report: Dysregulated immune system in children with autism: Beneficial effects of intravenous immune globulin on autistic characteristics J Autism Dev Disord 1996 26 439 452 8863094 10.1007/BF02172828

[B94] D'Souza Y Fombonne E Ward BJ No Evidence of Persisting Measles Virus in Peripheral Blood Mononuclear Cells From Children With Autism Spectrum Disorder Pediatrics 2006 118 1664 1675 17015560 10.1542/peds.2006-1262

[B95] Horvath K Perman JA Autistic disorder and gastrointestinal disease Curr Opin Pediatr 2002 14 583 587 12352252 10.1097/00008480-200210000-00004

[B96] Ashwood P Anthony A Torrente F Wakefield AJ Spontaneous mucosal lymphocyte cytokine profiles in children with autism and gastrointestinal symptoms: mucosal immune activation and reduced counter regulatory interleukin-10 J Clin Immunol 2004 24 664 673 15622451 10.1007/s10875-004-6241-6

[B97] Krigsman A Boris M Goldblatt A Stott C Clinical Presentation and Histologic Findings at Ileocolonoscopy in Children with Autistic Spectrum Disorder and Chronic Gastrointestinal Symptoms Autism Insights 2010 2010 1

[B98] Torrente F Ashwood P Day R Machado N Furlano RI Anthony A Davies SE Wakefield AJ Thomson MA Walker-Smith JA Murch SH Small intestinal enteropathy with epithelial IgG and complement deposition in children with regressive autism Mol Psychiatry 2002 7 334 375 382 11986981 10.1038/sj.mp.4001077

[B99] Horvath K Papadimitriou JC Rabsztyn A Drachenberg C Tildon JT Gastrointestinal abnormalities in children with autistic disorder J Pediatr 1999 135 559 563 10547242 10.1016/s0022-3476(99)70052-1

[B100] Wakefield AJ Anthony A Murch SH Thomson M Montgomery SM Davies S O'Leary JJ Berelowitz M Walker-Smith JA Enterocolitis in children with developmental disorders Am J Gastroenterol 2000 95 2285 2295 11007230 10.1111/j.1572-0241.2000.03248.x

[B101] D'Eufemia P Celli M Finocchiaro R Pacifico L Viozzi L Zaccagnini M Cardi E Giardini O Abnormal intestinal permeability in children with autism Acta Paediatr 1996 85 1076 1079 8888921 10.1111/j.1651-2227.1996.tb14220.x

[B102] Torrente F Anthony A Heuschkel RB Thomson MA Ashwood P Murch SH Focal-enhanced gastritis in regressive autism with features distinct from Crohn's and Helicobacter pylori gastritis Am J Gastroenterol 2004 99 598 605 15089888 10.1111/j.1572-0241.2004.04142.x

[B103] de Magistris L Familiari V Pascotto A Sapone A Frolli A Iardino P Carteni M De Rosa M Francavilla R Riegler G Alterations of the Intestinal Barrier in Patients With Autism Spectrum Disorders and in Their First-degree Relatives J Pediatr Gastroenterol Nutr 2010 51 418 424 410.1097/MPG.1090b1013e3181dcc1094a1095 20683204 10.1097/MPG.0b013e3181dcc4a5

[B104] Konstantynowicz J Porowski T Zoch-Zwierz W Wasilewska J Kadziela-Olech H Kulak W Owens SC Piotrowska-Jastrzebska J Kaczmarski M A potential pathogenic role of oxalate in autism Autism 2012 16 5 485 491 10.1016/j.ejpn.2011.08.004 21911305

[B105] Fasano A Intestinal zonulin: open sesame! Gut 2001 49 159 162 11454785 10.1136/gut.49.2.159 PMC1728387

[B106] Choi YJ Seelbach MJ Pu H Eum SY Chen L Zhang B Hennig B Toborek M Polychlorinated Biphenyls Disrupt Intestinal Integrity via NADPH Oxidase-Induced Alterations of Tight Junction Protein Expression Environ Health Perspect 2010 118 976 981 20299304 10.1289/ehp.0901751 PMC2920918

[B107] Jyonouchi H Geng L Ruby A Reddy C Zimmerman-Bier B Evaluation of an association between gastrointestinal symptoms and cytokine production against common dietary proteins in children with autism spectrum disorders J Pediatr 2005 146 605 610 15870662 10.1016/j.jpeds.2005.01.027

[B108] Knivsberg AM Reichelt KL HØien T NØdland M A Randomised, Controlled Study of Dietary Intervention in Autistic Syndromes Nutr Neurosci 2002 5 251 261 12168688 10.1080/10284150290028945

[B109] Pennesi CM Klein LC Effectiveness of the gluten-free, casein-free diet for children diagnosed with autism spectrum disorder: Based on parental report Nutr Neurosci 2012 15 85 91 22564339 10.1179/1476830512Y.0000000003

[B110] Whiteley P Haracopos D Knivsberg A-M Reichelt KL Parlar S Jacobsen J Seim A Pedersen L Schondel M Shattock P The ScanBrit randomised, controlled, single-blind study of a gluten- and casein-free dietary intervention for children with autism spectrum disorders Nutr Neurosci 2010 13 87 100 20406576 10.1179/147683010X12611460763922

[B111] Srinivasan P A review of dietary interventions in autism Ann Clin Psychiatr 2009 21 237 247 19917213

[B112] O’Mahony S Hyland N Dinan T Cryan J Maternal separation as a model of brain–gut axis dysfunction Psychopharmacology 2011 214 71 88 20886335 10.1007/s00213-010-2010-9

[B113] Ford AC Talley NJ Schoenfeld PS Quigley EMM Moayyedi P Efficacy of antidepressants and psychological therapies in irritable bowel syndrome: systematic review and meta-analysis Gut 2009 58 367 378 19001059 10.1136/gut.2008.163162

[B114] Goodhand JR Wahed M Rampton DS Management of stress in inflammatory bowel disease: a therapeutic option? Expert Review of Gastroenterology and Hepatology 2009 3 661 679 19929586 10.1586/egh.09.55

[B115] Blankstein U Chen J Diamant NE Davis KD Altered Brain Structure in Irritable Bowel Syndrome: Potential Contributions of Pre-Existing and Disease-Driven Factors Gastroenterology 2010 138 1783 1789 20045701 10.1053/j.gastro.2009.12.043

[B116] Amaral DG Schumann CM Nordahl CW Neuroanatomy of autism Trends Neurosci 2008 31 137 145 18258309 10.1016/j.tins.2007.12.005

[B117] Courchesne E Karns CM Davis HR Ziccardi R Carper RA Tigue ZD Chisum HJ Moses P Pierce K Lord C Unusual brain growth patterns in early life in patients with autistic disorder: an MRI study Neurology 2001 57 245 254 11468308 10.1212/wnl.57.2.245

[B118] Herbert MR Large brains in autism: the challenge of pervasive abnormality Neuroscientist 2005 11 417 440 16151044 10.1177/0091270005278866

[B119] Casanova M Buxhoeveden D J. G: Disruption in the inhibitory architecture of the cell minicolumn: implications for autism Neuroscientist 2003 9 496 507 14678582 10.1177/1073858403253552

[B120] Casanova MF van Kooten IAJ Switala AE van Engeland H Heinsen H Steinbusch HWM Hof PR Trippe J Stone J Schmitz C Minicolumnar abnormalities in autism Acta Neuropathol 2006 112 287 303 16819561 10.1007/s00401-006-0085-5

[B121] Casanova MF Switala AE Trippe J Fitzgerald M Comparative minicolumnar morphometry of three distinguished scientists Autism 2007 11 557 569 17947291 10.1177/1362361307083261

[B122] Baron-Cohen S Bolton P Wheelwright S Short L Mead G Smith A Scahill V Autism occurs more often in families of physicists, engineers, and mathematicians Autism 1998 2 296 301

[B123] Ploog B Stimulus Overselectivity Four Decades Later: A Review of the Literature and Its Implications for Current Research in Autism Spectrum Disorder J Autism Dev Disord 2010 40 1332 1349 20238154 10.1007/s10803-010-0990-2

[B124] Rubenstein JL Merzenich MM Model of autism: increased ratio of excitation/inhibition in key neural systems Genes Brain Behav 2003 2 255 267 14606691 10.1034/j.1601-183x.2003.00037.x PMC6748642

[B125] Just MA Cherkassky VL Keller TA Minshew NJ Cortical activation and synchronization during sentence comprehension in high-functioning autism: evidence of underconnectivity Brain 2004 127 1811 1821 15215213 10.1093/brain/awh199

[B126] Belmonte MK Cook EH Anderson GM Rubenstein JLR Greenough WT Beckel-Mitchener A Courchesne E Boulanger LM Powell SB Levitt PR Autism as a disorder of neural information processing: directions for research and targets for therapy Mol Psychiatr 2004 9 646 663 10.1038/sj.mp.4001499 15037868

[B127] Anderson MP Hooker BS Herbert MR Bridging from Cells to Cognition in Autism Pathophysiology: Biological Pathways to Defective Brain Function and Plasticity Am J Biochem Biotechnol 2008 4 167 176

[B128] Tuchman R Cuccaro M Alessandri M Autism and epilepsy: Historical perspective Brain Dev 2010 32 9 709 718 20510557 10.1016/j.braindev.2010.04.008

[B129] Frye RE Butler I Strickland D Castillo E Papanicolaou A Electroencephalogram discharges in atypical cognitive development J Child Neurol 2010 25 556 566 20299700 10.1177/0883073809344743 PMC2896832

[B130] Deth RC Molecular Origins of Human Attention 2003 Kluwer Academic Publishers

[B131] Rzhetsky A Wajngurt D Park N Zheng T Probing genetic overlap among complex human phenotypes Proc Nat Acad Sci 2007 104 11694 11699 17609372 10.1073/pnas.0704820104 PMC1906727

[B132] Reed M Thomas R Pavisic J James SJ Ulrich C Nijhout HF A mathematical model of glutathione metabolism Theor Biol Med Model 2008 5 8 18442411 10.1186/1742-4682-5-8 PMC2391141

[B133] Janusonis S Statistical distribution of blood serotonin as a predictor of early autistic brain abnormalities Theor Biol Med Model 2005 2 27 16029508 10.1186/1742-4682-2-27 PMC1199627

[B134] Rossignol DA Frye RE A review of research trends in physiological abnormalities in autism spectrum disorders: immune dysregulation, inflammation, oxidative stress, mitochondrial dysfunction and environmental toxicant exposures Mol Psychiatr 2011 17 4 389 401 10.1038/mp.2011.165 PMC3317062 22143005

[B135] Shoffner J Hyams L Langley GN Cossette S Mylacraine L Dale J Ollis L Kuoch S Bennett K Aliberti A Hyland K Fever Plus Mitochondrial Disease Could Be Risk Factors for Autistic Regression J Child Neurol 2010 25 429 434 19773461 10.1177/0883073809342128

[B136] Noble D Modeling the Heart–from Genes to Cells to the Whole Organ Science 2002 295 1678 1682 11872832 10.1126/science.1069881

[B137] BioUML Open Source Java Framework for SYstems Biologyhttp://www.biouml.org/index.shtml

[B138] Maus C Rybacki S Uhrmacher A Rule-based multi-level modeling of cell biological systems BMC Syst Biol 2011 5 166 22005019 10.1186/1752-0509-5-166 PMC3306009

[B139] Van Laar VS Berman SB The interplay of neuronal mitochondrial dynamics and bioenergetics: Implications for Parkinson's disease Neurobiol of Dis Available online 2 June 2012, ISSN 0969-9961 10.1016/j.nbd.2012.05.015(http://www.sciencedirect.com/science/article/pii/S096999611200201X PMC4015731 22668779

[B140] Parker RS Clermont G Systems engineering medicine: engineering the inflammation response to infectious and traumatic challenges J R Soc Interface 2010 7 989 1013 20147315 10.1098/rsif.2009.0517 PMC2880083

[B141] Ryan PB Thomas AB Cohen Hubal EA Jerome JC Thomas EM Using biomarkers to inform cumulative risk assessment Environ Health Perspect 2007 115 5 833 10.1289/ehp.9334 PMC1867975 17520075

[B142] Silins I Högberg J Combined Toxic Exposures and Human Health: Biomarkers of Exposure and Effect Int J Environ Res Publ Health 2011 8 629 647 10.3390/ijerph8030629 PMC3083662 21556171

[B143] Sexton K Hattis D Assessing Cumulative Health Risks from Exposure to Environmental Mixtures—Three Fundamental Questions Environ Health Perspect 2007 115 5 825 10.1289/ehp.9333 PMC1867955 17520074

[B144] Gonzalez A Stombaugh J Lozupone C Turnbaugh P Gordon J Knight R The mind-body-microbial continuum Dialogues Clin Neurosci 2011 13 55 62 21485746 10.31887/DCNS.2011.13.1/agonzalez PMC3139398

[B145] Jacobs DM Gaudier E Duynhoven J Vaughan EE Non-Digestible Food Ingredients, Colonic Microbiota and the Impact on Gut Health and Immunity: A Role for Metabolomics Curr Drug Metab 2009 10 41 54 19149512 10.2174/138920009787048383

[B146] Wolowczuk I Verwaerde C Viltart O Delanoye A Delacre M Pot B Grangette C Feeding Our Immune System: Impact on Metabolism Clin Dev Immunol 2008 10.1155/2008/639803 PMC2266987 18350123

[B147] Kohl P Noble D Systems biology and the virtual physiological human Mol Syst Biol 2009 5 292 19638973 10.1038/msb.2009.51 PMC2724980

[B148] Constantino JN Gruber CP Davis S Hayes S Passanante N Przybeck T The factor structure of autistic traits J Child Psychol Psychiatr 2004 45 719 726 10.1111/j.1469-7610.2004.00266.x 15056304

[B149] van Lang NDJ Boomsma A Sytema S de Bildt AA Kraijer DW Ketelaars C Minderaa RB Structural equation analysis of a hypothesised symptom model in the autism spectrum J Child Psychol Psychiatr 2006 47 37 44 10.1111/j.1469-7610.2005.01434.x 16405639

[B150] Tager-Flusberg H Joseph RM Identifying neurocognitive phenotypes in autism Phil Trans Roy Soc Lond Biol Sci 2003 358 303 314 12639328 10.1098/rstb.2002.1198 PMC1201482

[B151] Walsh P Elsabbagh M Bolton P Singh I In search of biomarkers for autism: scientific, social and ethical challenges Nat Rev Neurosci 2011 12 603 612 21931335 10.1038/nrn3113

[B152] Ratajczak HV Theoretical aspects of autism: biomarkers—a review J Immunot 2011 8 80 94 10.3109/1547691X.2010.538749 21299356

[B153] Chen L Xuan J Riggins R Clarke R Wang Y Identifying cancer biomarkers by network-constrained support vector machines BMC Syst Biol 2011 5 161 21992556 10.1186/1752-0509-5-161 PMC3214162

[B154] Bradstreet JJ Smith S Baral M Rossignol DA Biomarker-Guided Interventions of Clinically Relevant Conditions Associated with Autism Spectrum Disorders and Attention Deficit Hyperactivity Disorder Altern Med Rev 2010 15 15 32 20359266

[B155] Duda RO Hart PE Stork DG Pattern Classification 2001 2 John Wiley & Sons

[B156] Bezdek JC Pattern Recognition with Fuzzy Objective Function Algorithms 1981 Kluwer Academic Publishers

[B157] Handl J Knowles J Kell DB Computational cluster validation in post-genomic data analysis Bioinformatics 2005 21 3201 3212 15914541 10.1093/bioinformatics/bti517

[B158] Kohonen T Self-organizing maps 2000 Springer

[B159] Furey TS Cristianini N Duffy N Bednarski DW Schummer M Haussler D Support vector machine classification and validation of cancer tissue samples using microarray expression data Bioinformatics 2000 16 906 914 11120680 10.1093/bioinformatics/16.10.906

[B160] Brown MPS Grundy WN Lin D Cristianini N Sugnet CW Furey TS Ares M Haussler D Knowledge-based analysis of microarray gene expression data by using support vector machines Proc Natl Acad Sci 2000 97 262 267 10618406 10.1073/pnas.97.1.262 PMC26651

[B161] Bishop CM Pattern Recognition and Machine Learning 2006 Springer

[B162] D'Haeseleer P How does gene expression clustering work? Nat Biotech 2005 23 1499 1501 10.1038/nbt1205-1499 16333293

[B163] Verleysen M François D Cabestany J, Prieto A, Sandoval F The Curse of Dimensionality in Data Mining and Time Series Prediction Computational Intelligence and Bioinspired Systems Lecture Notes in Computer Science 2005 Heidelberg: Springer Berlin 85 125

[B164] Gheyas IA Smith LS Feature subset selection in large dimensionality domains Pattern Recogn 2010 43 5 13

[B165] Little RJ Rubin DB Statistical Analysis with Missing Data 2002 2 NJ: Wiley-Interscience, Hoboken

[B166] Randolph-Gips M A New Neural Network to Process Missing Data without Imputation Seventh International Conference on Machine Learning and Applications; Dec. 11-13 2008 CA USA: San Diego 756 762

[B167] Naik US Gangadharan C Abbagani K Nagalla B Dasari N Manna SK A Study of Nuclear Transcription Factor-Kappa B in Childhood Autism PLoS ONE 2011 6 2 7 10.1371/journal.pone.0019488 PMC3090385 21573053

[B168] Pearl J An Introduction to Causal Inference Int J Biostat 2010 6 2 10.2202/1557-4679.1203 PMC2836213 20305706

[B169] Tiemann C Vanlier J Hilbers P van Riel N Parameter adaptations during phenotype transitions in progressive diseases BMC Syst Biol 2011 5 174 22029623 10.1186/1752-0509-5-174 PMC3354367

[B170] Ahmed S Ahameethunisa A Santosh W Chakravarthy S Kumar S Systems biological approach on neurological disorders: a novel molecular connectivity to aging and psychiatric diseases BMC Syst Biol 2011 5 6 21226925 10.1186/1752-0509-5-6 PMC3033822

[B171] Wolstenholme JT Edwards M Shetty SRJ Gatewood JD Taylor JA Rissman EF Connelly JJ Gestational Exposure to Bisphenol A Produces Transgenerational Changes in Behaviors and Gene Expression Endocrinology 2012 153 3828 3838 22707478 10.1210/en.2012-1195 PMC3404345

[B172] Garrecht M Austin DW The plausibility of a role for mercury in the etiology of autism: a cellular perspective Toxicol Environ Chem 2011 93 1251 1273 22163375 10.1080/02772248.2011.580588 PMC3173748

[B173] Nickerson K Environmental Contaminants in Breast Milk J Midwifery Women Health 2006 51 26 34 10.1016/j.jmwh.2005.09.006 16399607

[B174] Shandley K Austin DW Ancestry of Pink Disease (Infantile Acrodynia) Identified as a Risk Factor for Autism Spectrum Disorders J Toxicol Environ Health Part A 2011 74 1185 1194 21797771 10.1080/15287394.2011.590097 PMC3173747

[B175] Jirtle RL Skinner MK Environmental epigenomics and disease susceptibility Nat Rev Genet 2007 8 253 262 17363974 10.1038/nrg2045 PMC5940010

[B176] Oh W Gelardi NL Cha CJ The cross-generation effect of neonatal macrosomia in rat pups of streptozotocin-induced diabetes Pediatr Res 1991 29 606 610 1866217 10.1203/00006450-199106010-00016

[B177] Boloker J Gertz SJ Simmons RA Gestational diabetes leads to the development of diabetes in adulthood in the rat Diabetes 2002 51 1499 1506 11978648 10.2337/diabetes.51.5.1499

[B178] Sakoe H Chiba S Dynamic programming algorithm optimization for spoken word recognition Acoust Speech Signal Process IEEE Trans 1978 26 43 49

[B179] Dubinsky MC Lin Y-C Dutridge D Picornell Y Landers CJ Farrior S Wrobel I Quiros A Vasiliauskas EA Grill B Serum Immune Responses Predict Rapid Disease Progression among Children with Crohn's Disease: Immune Responses Predict Disease Progression Am J Gastroenterol 2006 101 360 367 16454844 10.1111/j.1572-0241.2006.00456.x PMC2259248

[B180] Liebregts T Adam B Bredack C Röth A Heinzel S Lester S Downie-Doyle S Smith E Drew P Talley NJ Holtmann G Immune Activation in Patients With Irritable Bowel Syndrome Gastroenterology 2007 132 913 920 17383420 10.1053/j.gastro.2007.01.046

[B181] Mayer EA Gut feelings: the emerging biology of gut–brain communication Nat Rev Neurosci 2011 12 453 466 21750565 10.1038/nrn3071 PMC3845678

[B182] Su L Shen L Clayburgh DR Nalle SC Sullivan EA Meddings JB Abraham C Turner JR Targeted Epithelial Tight Junction Dysfunction Causes Immune Activation and Contributes to Development of Experimental Colitis Gastroenterology 2009 136 551 563 19027740 10.1053/j.gastro.2008.10.081 PMC2712351

[B183] James SJ Zimmerman AW Oxidative Stress and the Metabolic Pathology of Autism Autism 2008 NJ: Humana Press, Totowa

